# Influence of Restorative Material Properties on Dentin Stress Distribution: A 3D Finite Element Analysis of Bioflx and Zirconia Crowns

**DOI:** 10.3390/jfb17050226

**Published:** 2026-05-04

**Authors:** Enes Bardakci, Guldeste Aydin, Peris Celikel

**Affiliations:** 1 Department of Pediatric Dentistry, Faculty of Dentistry, Harran University, Sanliurfa 63290, Türkiye; guldesteaydin@harran.edu.tr; 2 Department of Pedodontics, Faculty of Dentistry, Ataturk University, Erzurum 25040, Türkiye; peris.celikel@atauni.edu.tr

**Keywords:** Bioflx crown, zirconia crown, pediatric dentistry, pulpotomy, dentin, finite element analysis, stress distribution

## Abstract

Aim: The aim of this study is to evaluate the effect of restorative crown materials with different elastic moduli on stress distribution in dentin and supporting tissues of pulpotomized primary anterior teeth under multi-directional loading conditions using the three-dimensional finite element analysis method. Materials and Methods: A three-dimensional model of a maxillary primary central incisor was created based on anatomical data. A clinical pulpotomy scenario was simulated using mineral trioxide aggregate (MTA) and resin-modified glass ionomer cement. Three models were analyzed: healthy tooth (control), Bioflx crown, and prefabricated zirconia crown. Frontal, oblique, and vertical loads were applied to represent functional and traumatic conditions. von Mises and principal stress distributions in the crown, dentin, and supporting tissues were evaluated. Results: In the prefabricated zirconia crown group, higher von Mises stress values were observed under all loading conditions, with significant stress concentrations particularly in the cervical region. In contrast, the Bioflx crown group exhibited lower stress values and a more homogeneous stress distribution. While the stress patterns in the Bioflx group were found to be closer to those of the control group, more localized stress accumulation was observed in the zirconia crowns. No significant differences were observed between the groups in the bone tissue. Conclusions: The elastic modulus of restorative materials plays a decisive role in the stress transfer mechanism. It is believed that materials with dentin-like mechanical properties may provide a more balanced and physiological stress distribution. Multi-directional loading analysis highlights the importance of evaluating the biomechanical behavior of restorative materials under more realistic conditions. Further advanced experimental and clinical studies are needed to clinically validate these findings.

## 1. Introduction

Early childhood caries (ECC) is one of the most common diseases during childhood worldwide and is recognized as a significant public health issue [1,2]. Since primary teeth, particularly those in the anterior region, are of critical importance in terms of aesthetics, speech, and masticatory functions, advanced caries lesions in these teeth can negatively affect both children’s oral health and quality of life [3,4]. The progression of caries lesions leads to pulp involvement, and in such cases, vital pulp treatments such as pulpotomy become necessary to preserve the tooth’s function [5].

The long-term success of pulpotomy treatment is directly related to the properties of the restorative material used. For this purpose, prefabricated crowns are frequently preferred for primary teeth that have undergone pulpotomy due to their superior sealing properties and mechanical strength [6]. Prefabricated zirconia crowns have found widespread clinical use due to their high aesthetic properties, biocompatibility, and mechanical strength. However, zirconia is characterized by a high elastic modulus and inherent rigidity, which may lead to increased stress transmission to the underlying dentin and supporting tissues. In addition, the technique sensitivity and the requirement for greater tooth structure reduction are considered important clinical limitations [7].

Bioflx crowns, one of the new-generation restorative materials developed in recent years, stand out for their flexible structure, ease of adaptation, and minimal preparation requirements. These crowns are composed of high-performance polymer-based materials designed to exhibit a lower elastic modulus compared to conventional rigid systems. This characteristic enables them to absorb and dissipate functional forces more effectively, potentially reducing stress concentration within dental tissues. The fact that these materials have a lower elastic modulus suggests they may offer biomechanical advantages in terms of their potential to absorb applied forces and distribute them more evenly. This biomechanical advantage may have important clinical implications, particularly in reducing stress concentration at the dentin–restoration interface and improving the longevity of restorations in primary teeth [8].

One of the primary goals in the development of restorative materials is to mimic the biomechanical behavior of dentin and preserve dentin integrity. Natural dentin is able to absorb applied forces and distribute stresses more homogeneously due to its relatively low modulus of elasticity. In contrast, mechanical property differences between dentin and restorative materials can lead to stress concentration, particularly at the dentin–restoration interface, which may increase the risk of structural failure over the long term. Mechanical mismatch between dentin and restorative materials not only causes stress concentration but also directly affects interface stability and restoration success over the long term [9,10].

Finite element analysis (FEA) is a powerful numerical method widely used to evaluate stress distributions in dental tissues and restorative materials. However, the majority of studies in the literature have focused on posterior teeth or traditional restorative materials [11,12]. In particular, studies evaluating the biomechanical behavior of new-generation flexible crown systems in pulpotomized primary anterior teeth are limited, and the potential of these materials to create dentin-like stress distributions has not been sufficiently demonstrated [13,14].

Previous finite element studies have primarily focused on stress distribution in posterior teeth or conventional restorative materials, and therefore may not adequately represent the biomechanical behavior of flexible crown systems in primary anterior teeth [15,16,17]. In addition, the effect of multi-directional loading conditions on stress transfer mechanisms in these materials remains insufficiently explored.

Based on this information, the aim of our study is to comparatively evaluate the stress distribution patterns of prefabricated zirconia crowns and Bioflx crowns under different loading conditions in upper primary central incisors treated with mineral trioxide aggregate (MTA) pulpotomy, using the three-dimensional finite element analysis method. Additionally, the study aims to investigate the effect of restorative materials with different elastic moduli on stress transfer mechanisms in dentin and surrounding tissues, and to elucidate the role of material selection in achieving dentin-like biomechanical behavior. In this context, another objective of the study is to contribute to the identification of a restorative material that exhibits behavior closer to that of dentin. The null hypothesis of the study is that there is no significant difference in the stress distribution patterns on dentin and surrounding tissues between restorative crown materials with different elastic moduli (Bioflx and prefabricated zirconia crowns) in maxillary primary central incisors treated with MTA pulpotomy.

## 2. Materials and Methods

This study was designed using the FEA method to evaluate the stress distribution of different restorative crown materials under traumatic forces in maxillary primary central incisors treated with MTA pulpotomy. Analyses were performed under static and linear elastic conditions.

### 2.1. Modeling Procedure

**Figure 1:**
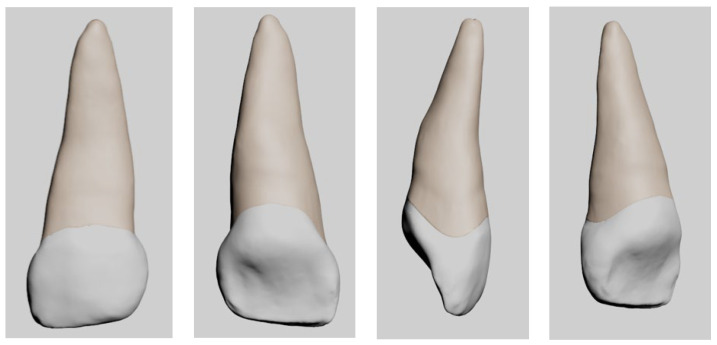
Three-dimensional geometric model of a healthy maxillary primary central incisor used as the baseline structure for finite element analysis.

A three-dimensional (3D) geometric model of the maxillary primary central incisor was created using [18] based on anatomical averages and micro-computed tomography (micro-CT) data to ensure morphological accuracy. The initial model represents a healthy maxillary primary central incisor (Figure 1).

The geometric modeling process was performed using Blender software (version 4.2, Blender Foundation, Netherlands), with dental tissues such as enamel, dentin, and pulp defined in detail. The created models were exported in .stl and .obj formats, then converted into a solid model using FreeCAD (version 1.0, open-source software; FreeCAD Community) and converted to the appropriate. step format for analysis. In the final stage, the models were imported into 3DEXPERIENCE SIMULIA (Dassault Systèmes, France; R2025x) software to perform finite element analyses.

All materials were assumed to be linearly elastic, homogeneous, and isotropic. During the meshing process, the C3D4 (four-node tetrahedral element) type was used.

To simulate a clinical pulpotomy scenario, an access cavity 4 mm deep was prepared in the model and extended 1 mm below the coronal pulp–enamel–cementum junction. Subsequently, a 3 mm-thick layer of MTA was applied, followed by a resin-modified glass ionomer cement. Two different restorative scenarios were modeled:

- Prefabricated zirconia crown
- Bioflx crown

The crowns were designed to be 0.5 mm thick in the cervical region and 1.0 mm thick in the incisal region and were integrated into the model with a 0.2 mm-thick cement layer.

### 2.2. Supporting Structures

To simulate the surrounding supporting tissues, the periodontal ligament (PDL), cortical bone, and trabecular bone structures were included in the model. Cortical bone was modeled with a 2.0 mm offset from the exterior of the tooth structure; trabecular bone was modeled 1.5 mm inside the cortical layer [19]. The PDL was modeled with a homogeneous thickness of 0.2 mm along the root surface to reflect the average dimensions of primary teeth.

### 2.3. Restorative Scenarios

**Figure 2:**
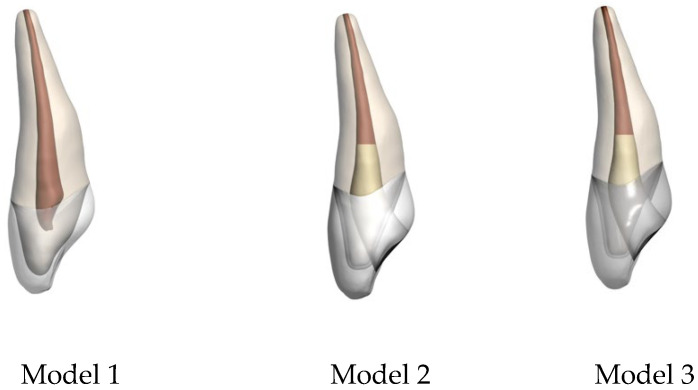
Finite element models of upper central incisors restored using different crown materials following MTA pulpotomy.

Three different tooth models were prepared to simulate clinical treatment following MTA pulpotomy (Figure 2):

- **Model 1:** Control (healthy primary anterior tooth without restoration)
- **Model 2:** MTA pulpotomy + resin-modified glass ionomer cement applied as the base material + Bioflx crown
- **Model 3:** MTA pulpotomy + resin-modified glass ionomer cement as the base material + prefabricated zirconia crown

The mechanical properties (modulus of elasticity and Poisson’s ratio) of all modeled tissues and materials were assigned based on values reported in the literature [20] (Table 1).

### 2.4. Mesh Structure and Boundary Conditions

The models were created using a high-density mesh structure consisting of approximately 299,734 nodes and 1,222,868 elements [21]. Mesh density was increased in the cervical region, the PDL interface, and the restoration–tooth interface areas to optimize analysis accuracy.

The outer surfaces of the cortical bone were fixed in all axes, and all contact surfaces were defined as “bonded.” This approach assumes that no separation occurs under load.

These assumptions, including bonded contact conditions, linear elastic behavior, and isotropic material properties, were adopted to simplify the model and are consistent with commonly used approaches in dental finite element studies [22,23].

### 2.5. Load Scenarios

**Figure 3:**
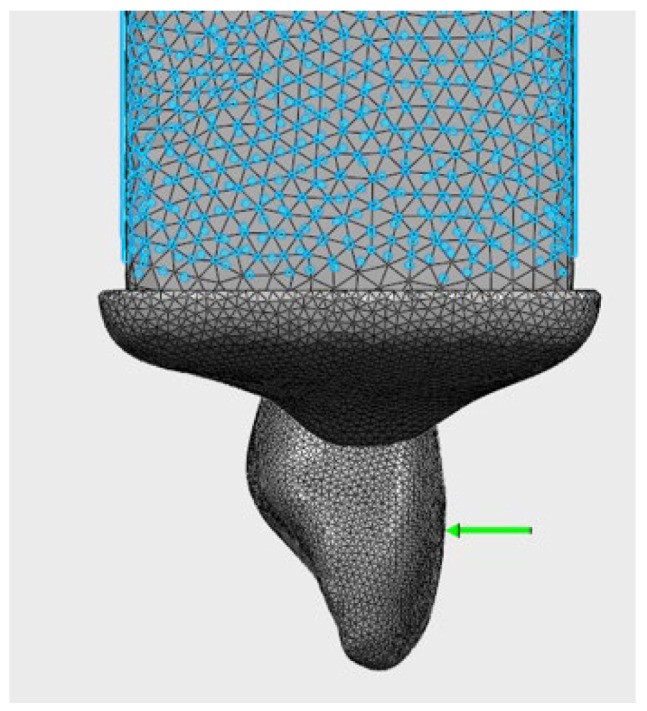
Stress distribution under frontal loading (82 N) applied perpendicular to the labial surface.

**Figure 4:**
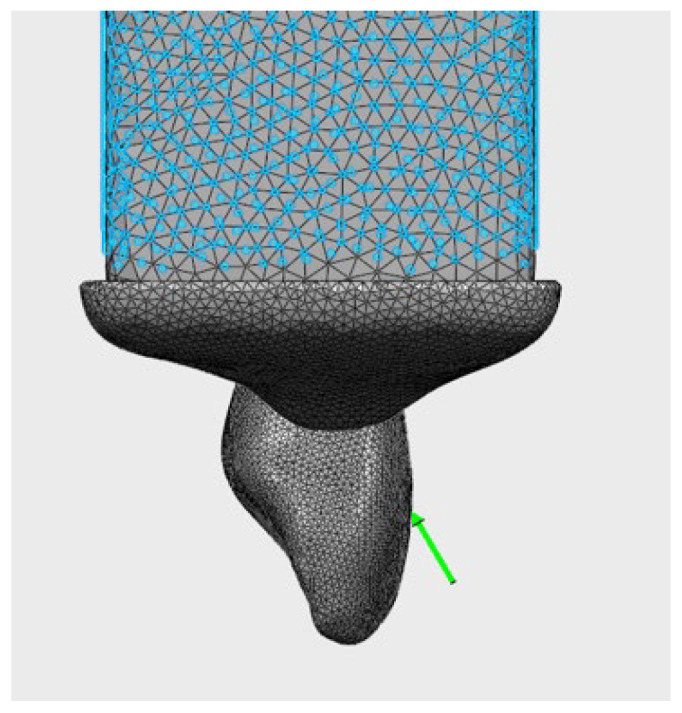
Stress distribution under oblique loading (166 N) applied at a 45° angle.

**Figure 5:**
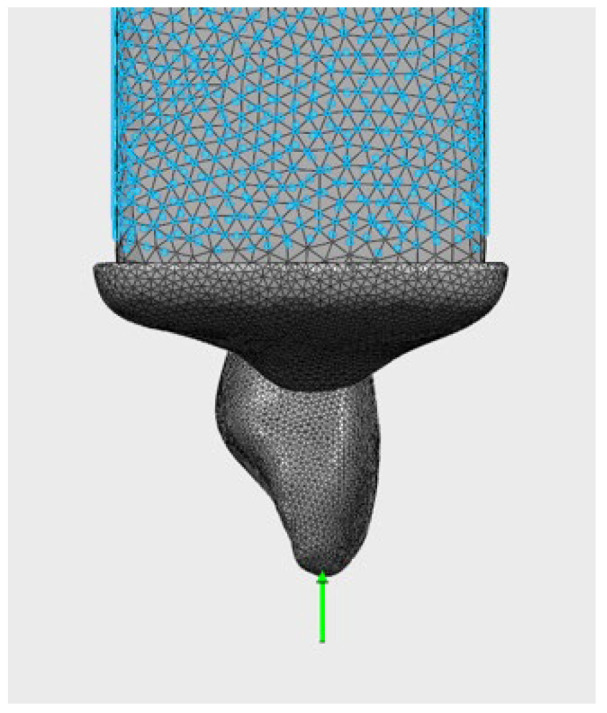
Stress distribution under vertical loading (171 N) applied along the long axis of the tooth.

Three different loading scenarios were applied:

- Frontal loading: 82 N (perpendicular to the labial surface) (Figure 3)
- Oblique loading: 166 N (at a 45° angle) (Figure 4)
- Vertical load: 171 N (along the long axis) (Figure 5)

These loads were determined based on the literature to simulate trauma and functional forces on the anterior teeth [24].

### 2.6. Model Validation

A mesh convergence analysis was performed to evaluate the accuracy of the model. Coarse, medium, and fine mesh structures were compared, and the fact that the difference between the maximum stress values obtained was less than 5% demonstrated that the model has sufficient accuracy.

Additionally, the model geometry and loading conditions were determined to be consistent with studies in the literature, and the resulting stress distribution patterns were accepted as biomechanically valid [24].

In addition, the modeling approach and boundary conditions were designed in accordance with previously published finite element studies in dental biomechanics, ensuring methodological consistency and supporting the validity of the simulation results.

### 2.7. Statistical Analysis

Since this study is based on finite element analysis, the results were obtained through deterministic numerical simulations rather than experimental or clinical data sampling. Therefore, conventional statistical analyses were not applicable. Instead, comparisons were made based on the magnitude and distribution patterns of stress values obtained under different loading conditions.

## 3. Results

### 3.1. Stresses Developed in the Crown Material

Table 2 shows the von Mises stress values obtained for the different crown materials under various loading conditions. According to the results obtained for crown materials, the highest von Mises stress values were observed in the prefabricated zirconia crown group under all loading scenarios. Specifically, under frontal loading, the maximum von Mises stress value in zirconia crowns was determined to be 237.0 MPa. In contrast, significantly lower stress values were obtained in the Bioflx crown group under all loading conditions, with von Mises stress values ranging from 8.30 to 13.4 MPa. The values obtained in the control group were generally lower than those in the zirconia group but higher than those in the Bioflx group.

### 3.2. Stresses Developed in the Tooth

When examining the von Mises stresses in the dentin, the highest stress values were observed in all groups under frontal loading. Values of 56.4 MPa were obtained in the control group, 57.8 MPa in the zirconia group, and 58.5 MPa in the Bioflx group under frontal loading. Under oblique loading, lower stress values were determined in all groups, with the lowest value found in the Bioflx group at 25 MPa. Under vertical loading, moderate stress values were observed in all groups (Table 3).

### 3.3. Stresses in Cortical Bone

The maximum principal stress values observed in cortical bone were similar across groups. In all groups, the lowest stress values were obtained under frontal loading, while higher values were observed under oblique and vertical loading conditions. In the control group, the maximum principal stress value was 23.0 MPa under vertical loading, representing the highest level. In the Zircon and Bioflx groups, a similar stress distribution was observed under oblique and vertical loading. When examining the minimum principal stress values, no significant difference was observed between the groups (Table 4).

### 3.4. Von Mises Stresses in the Tooth

**Figure 6:**
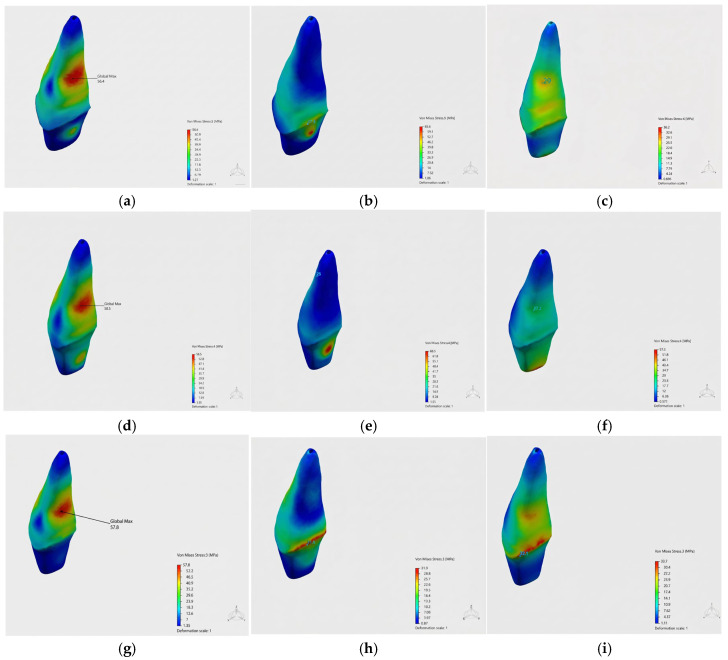
Von Mises stress distributions in dentin: (**a**–**c**) control group, (**d**–**f**) Bioflx crown group, and (**g**–**i**) prefabricated zirconia crown group under frontal, oblique, and vertical loading conditions.

Von Mises stress distributions in dentin were evaluated according to different loading scenarios and restorative materials (Figure 6).

While higher stress values were observed in all groups under frontal loading, lower stress values were determined under oblique and vertical loading conditions. In the control group, stress was observed to concentrate in the root mid-third region under frontal loading.

It was determined that stress distribution was generally more homogeneous in the Bioflx crown model, and lower stress concentrations were observed, particularly under oblique loading.

In the prefabricated zirconia crown model, stress was found to be concentrated in more localized areas, with a distinct accumulation of stress particularly in the cervical region.

Figure 6 shows the stress distributions in dentin under frontal, oblique, and vertical loading scenarios for the control (a–c), Bioflx (d–f), and zirconia (g–i) crown groups, represented by a color scale.

### 3.5. Von Mises Stress Distributions in Crown Materials

The von Mises and principal stress distributions in the crown materials were evaluated under different loading conditions (Figure 3, Figure 4 and Figure 5). Figure 3 shows the von Mises and principal stress distributions for the control group, Figure 4 for the Bioflx crown group, and Figure 5 for the zirconia crown group under frontal, oblique, and vertical loading conditions.

In the control group, stress distribution was observed at lower levels and was relatively homogeneous.

In the Bioflx crown group, the stress distribution was observed to be more widespread across the crown surface and did not show a distinct localization. In the prefabricated zirconia crown group, however, stress was observed to be concentrated particularly in the cervical region and specific localized areas.

In the comparison of different crown materials, higher von Mises stress values were obtained in the zirconia crown group under all loading conditions. In the Bioflx crown group, lower stress values were observed in all loading scenarios. The stress values obtained in the control group were intermediate compared to both restorative groups.

**Figure 7:**
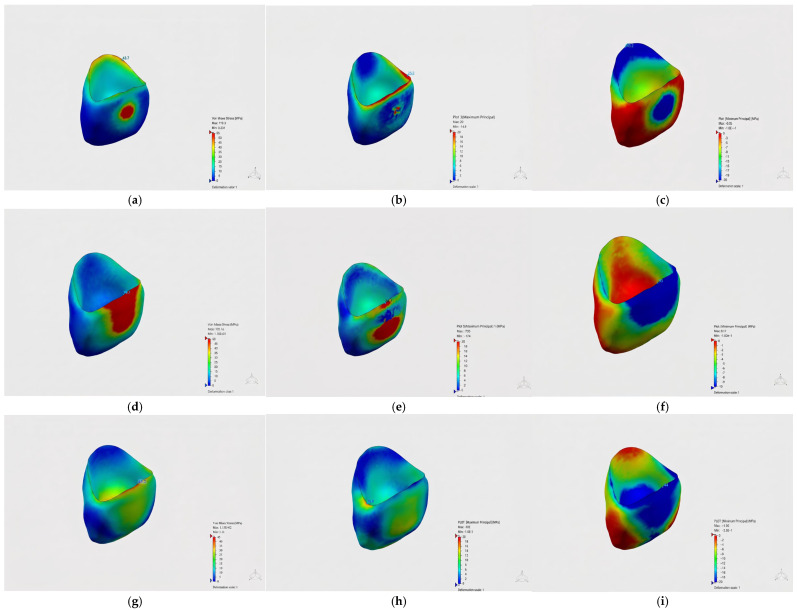
Stress distributions in crown materials for the control group under frontal (**a**–**c**), oblique (**d**–**f**), and vertical (**g**–**i**) loading conditions.

**Figure 8:**
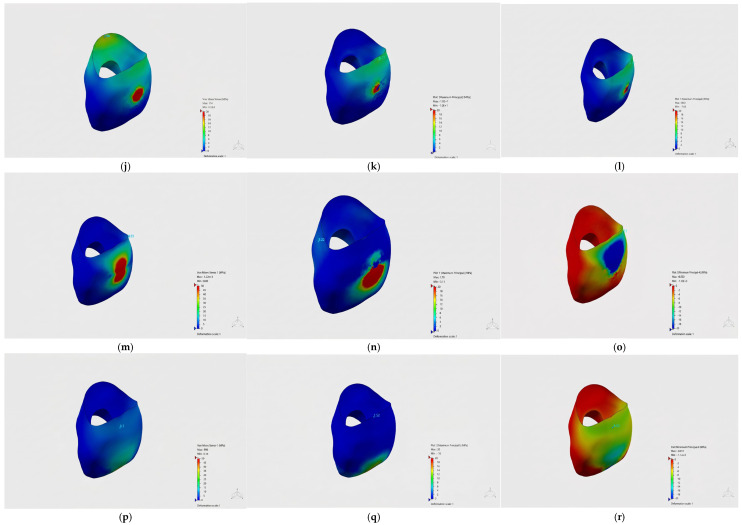
Stress distributions in crown materials for the Bioflx crown group under frontal (**j**–**l**), oblique (**m**–**o**), and vertical (**p**–**r**) loading conditions.

**Figure 9:**
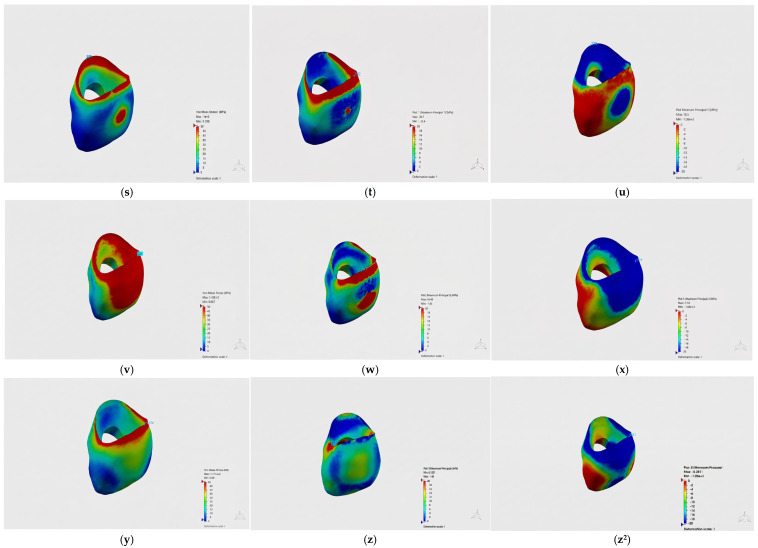
Stress distributions in crown materials for the prefabricated zirconia crown group under frontal (**s**–**u**), oblique (**v**–**x**), and vertical (**y**–**z^2^**) loading conditions.

Detailed stress distribution patterns for each material group are further illustrated in Figure 7, Figure 8 and Figure 9, where Figure 7 corresponds to the control group, Figure 8 to the Bioflx crown group, and Figure 9 to the zirconia crown group under all loading scenarios.

### 3.6. Maximum and Minimum Principal Stresses in Cortical Bone

The maximum and minimum principal stress values in cortical bone were evaluated under different loading conditions.

Lower stress values were observed in all groups under frontal loading, while higher stress values were obtained under oblique and vertical loading conditions.

No significant difference was observed between the groups at the cortical bone level.

## 4. Discussion

To the best of our knowledge, this study is one of a limited number of studies that comparatively evaluate the biomechanical behavior of Bioflx and zirconia crowns in pulpotomised primary anterior teeth under different loading directions (frontal, oblique, and vertical). The findings of this study indicate that the choice of restorative material significantly affects stress distribution on dentin and surrounding tissues in pulpotomised primary incisors. Accordingly, the null hypothesis of the study was rejected. Specifically, when examining crown materials, higher von Mises stress values and pronounced stress concentrations were observed in the prefabricated zirconia crown group under all loading conditions, whereas the Bioflx crown group exhibited lower stress values and a more homogeneous distribution. Based on these findings, it has been established that the elastic modulus of the restorative material is a fundamental parameter in determining the load transfer mechanism. This finding can be explained by the mismatch in elastic modulus between restorative materials and dentin. Materials with high elastic modulus, such as zirconia, tend to transfer stresses directly to dentin, leading to localized stress concentration [22]. In contrast, materials with lower elastic modulus, such as Bioflx, absorb and dissipate forces more effectively, resulting in a more homogeneous stress distribution.

The high von Mises stress values and the concentration of stress in specific regions observed in the zirconia crown group indicate that this material exhibits rigid behavior due to its high elastic modulus. This situation can be explained by the fact that loads are transmitted directly to the dentin and supporting tissues rather than being absorbed within the material. Similarly, the literature reports that restorative materials with high elastic modulus can increase stress concentration [25]. In contrast, lower stress values and a more homogeneous distribution were observed in the Bioflx crown group. This finding demonstrates that materials with lower elastic modulus can absorb loads and distribute them more evenly. Previous studies have also suggested that materials with a lower elastic modulus can create a more physiological stress distribution on dentin [26,27].

When evaluating stress distributions in dentin, it was observed that the values obtained in the Bioflx crown group were closer to those of the control group. This suggests that the material in question may exhibit dentin-like biomechanical behavior. In contrast, the concentration of stress in more localized areas in zirconia crowns can be considered a mechanism that may lead to stress accumulation at the dentin–restoration interface. The relatively similar dentin stress values observed across different restorative material groups may be explained by the intrinsic mechanical properties of dentin. Due to its viscoelastic nature and intermediate elastic modulus, dentin may act as a stress-regulating structure, distributing applied forces more uniformly and reducing the influence of differences in crown material properties.

When the effects of loading types were examined, it was determined that the highest stress values occurred under frontal loading in all groups. This indicates that anterior teeth are particularly sensitive to traumatic forces. However, studies in the literature also report that oblique loads generate higher stress, supporting the decisive role of loading direction on stress distribution [28,29].

From the perspective of bone tissue, the study noted that the effect of the restorative material on bone stress distribution is limited [27,30]. The results of the study also support this view. However, the key point here is that despite similar stress values in the bone, the different stress patterns formed at the tooth and crown levels may have indirect effects on the bone over the long term. Therefore, it has been observed that selecting a material based solely on bone stress values is insufficient.

One of the notable contributions of this study is the direct comparison of the biomechanical behavior of Bioflx crowns, which have been studied to a limited extent in the literature, with zirconia crowns, which are widely used in clinical practice. The findings suggest that, despite their high mechanical strength, zirconia crowns may not be the most suitable option from a biomechanical perspective in every clinical situation. In contrast, the more homogeneous stress distribution and lower stress values observed in the Bioflx crown group suggest that this material may exhibit dentin-like biomechanical behavior. In this context, one of the most important findings of this study is that restorative materials should be evaluated not only in terms of mechanical strength but also in terms of biomechanical compatibility with dentin. The data obtained indicate that materials with dentin-like mechanical properties may provide a more balanced stress distribution. This study provides a biomimetic perspective for restorative material selection in pediatric dentistry.

From a clinical perspective, these findings suggest that restorative materials with lower elastic modulus, such as Bioflx, may provide a more favorable stress distribution by reducing stress concentration in dentin. In contrast, stiffer materials such as zirconia may increase localized stress, which could influence long-term structural behavior.

Certain limitations must be considered when evaluating the study’s findings. This study employed a static loading protocol, and the dynamic and repetitive forces observed in clinical settings were not included in the model. Additionally, the finite element model assumed linear elastic, homogeneous, and isotropic material behavior, as well as perfectly bonded interfaces. In clinical conditions, dental tissues and restorative materials exhibit anisotropic and viscoelastic properties, and interfacial bonding may not be ideal. Microscopic voids, cementation defects, and the complex structure of biological tissues were also not represented in the model. Therefore, the stress values obtained in this study may differ from those observed under real clinical conditions and should not be directly generalized. However, these simplifications are widely accepted in finite element studies and allow for controlled comparison between different restorative materials.

## 5. Conclusions

This study demonstrated that Bioflx crowns, due to their low elastic modulus, absorb loads and provide a more homogeneous stress distribution, whereas rigid zirconia crowns generate higher stress values and localized stress concentrations. These findings highlight that the elastic modulus of restorative materials plays a decisive role in the stress distribution mechanism.

In this context, it is understood that in the selection of restorative materials, not only aesthetics and mechanical durability but also biomechanical compatibility with dentin must be considered. In particular, it is believed that materials with dentin-like mechanical properties may provide a more physiological stress distribution. However, due to the in vitro and computational modeling-based nature of this study, the direct generalization of the findings to the clinical setting is limited. Therefore, advanced in vitro and prospective clinical studies are needed to evaluate the long-term clinical success, wear resistance, and cementation stability of Bioflx crowns. These findings may assist clinicians in selecting restorative materials based not only on esthetic and mechanical considerations but also on their biomechanical compatibility with dental tissues.

## Figures and Tables

**Table 1:** Elastic modulus and Poisson’s ratio of modeled dental tissues and restorative materials used in finite element analysis.

Material/Tissue	Modulus of Elasticity (MPa)	Poisson’s Ratio
Deciduous tooth enamel	80,349	0.33
Dental dentin	19,890	0.31
Pulp	30	0.45
Periodontal ligament	50	0.49
Bioflx crown	5030	0.39
Prefabricated zirconia crown	205,000	0.19
Mineral Trioxide Aggregate (MTA)	11,700	0.31
Cortical bone	13,700	0.30
Cancellous (spongy) bone	1370	0.30
Resin-modified glass ionomer cement	3700	0.30

**Table 2:** Von Mises, maximum, and minimum principal stress values in crown materials.

(Crown) Groups	Loading Type	Max. Principal Stress (MPa)	Min. Principal Stress (MPa)	Von Mises Stress (MPa)
Control	Oblique	24.1	−93.0	98.7
	Vertical	16.9	−44.0	45.2
	Frontal	25.2	−43.2	43.7
Zircon	Oblique	58.1	−234.0	221.0
	Vertical	27.7	−201.0	174.0
	Frontal	202.0	−273.0	237.0
Bioflx	Oblique	3.22	−11.0	8.55
	Vertical	1.52	−8.62	8.30
	Frontal	9.77	−14.2	13.4

**Table 3:** Von Mises, maximum, and minimum principal stress values in dentin.

(Dentin) Group	Loading Type	Max. Principal Stress (MPa)	Min. Principal Stress (MPa)	Von Mises Stress (MPa)
Control	Oblique	8.11	−48.2	47.3
	Vertical	3.74	−31.0	29.0
	Frontal	61.1	−55.5	56.4
Zircon	Oblique	6.39	−27.9	31.5
	Vertical	6.13	−36.1	32.1
	Frontal	60.2	−62.0	57.8
Bioflx	Oblique	6.40	−27.4	25
	Vertical	2.45	−29.5	27.2
	Frontal	9.77	−53.9	58.5

**Table 4:** Maximum and minimum principal stress values (MPa) in cortical bone under different loading conditions for each group.

(Bone) Group	Loading Type	Max. Principal Stress (MPa)	Minimum Principal Stress (MPa)
Control	Oblique	20.2	−15.3
	Vertical	23.0	−11.1
	Frontal	14.7	−17.0
Zircon	Oblique	21.4	−17.0
	Vertical	20.8	−13.1
	Frontal	14.2	−14.3
Bioflx	Oblique	21.0	−16.1
	Vertical	21.0	−13.2
	Frontal	14.0	−14.3

## Data Availability

The original contributions presented in this study are included in the article. Further inquiries can be directed to the corresponding author.
